# Stronger tailwinds for efforts in endometriosis: let us explore new horizons

**DOI:** 10.1007/s00404-022-06869-7

**Published:** 2022-12-20

**Authors:** Bernhard Krämer

**Affiliations:** University Hospital for Women, Tuebingen, Germany

## What does this study add to the clinical work

**Table:** 

Endometriosis is getting more attention considering the high number of affected females worldwide. New scientific efforts and political directives are likely to change current practice.	

Thanks to a great interdisciplinary achievement, endometriosis is currently gaining more and broader attention—it is a development in the right direction that awareness campaigns as well as substantial basic and clinical research have shifted this condition into the limelight of gynaecology! Finally, political leaders are beginning to realize that endometriosis plays a role in women`s health and productivity in many fields: the governments of France and Germany recently announced initiatives to financially promote and support further projects focusing on endometriosis. This is an opportunity to increase the number of activities that ultimately lead to evidence-based results for the treatment and management of this chronic disease—and there is still a lot of work to do.

Endometriosis affects about 10% of women in their reproductive age, and to date we still treat the main problems with infertility and pain being the lead symptoms. Yet, the cause of the disease remains unclear. Several theories exist on how fertility is negatively affected and how the inhomogeneous pain sensations in relation to different endometriosis stages could be explained; however, the main trigger factors of these seemingly multifactorial pathways are not fully understood.

Mutations in endometriotic lesions can be detected, but their clinical role and origin are still not fully clear. Endometriosis is classified as a benign disease, but it shares key features with cancers such as resistance to apoptosis and stimulation of angiogenesis. It has been shown that deep infiltrating endometriosis rarely undergoes malignant transformation, still it harbours recurrent somatic mutations. According to recent publications in the field, endometriosis represents an oligoclonal disease with dissemination likely to consist of multiple epithelial clones travelling together. Consequently, the present anatomically defined classification does appropriately consider the aetiology of the condition. An updated classification should recognize genomic and molecular features with the possibility of introducing personalized diagnosis and care.

Clinically, the updated #ENZIAN classification has brought a valuable improvement where a common language is used to describe the location and the extent of the lesions in the different compartments of the pelvis and beyond. With ultrasound playing a major role as a non-invasive, easily available and accurate tool in obstetrics and gynaecology, this technique is further becoming a cornerstone of the preoperative diagnosis of endometriosis. It has been demonstrated that deep infiltrating lesions of the bowel and the urinary tract can be well detected sonongraphically—this has a great impact on the planning of the surgical strategy and the conservation of organs that are relevant for reproduction or assisted techniques. It is desirable that surgeons who operate on endometriosis are able to perform these ultrasound examinations themselves; however, there is a learning curve to pass until smaller lesions can be diagnosed reliably. It is therefore necessary to promote training and teaching in this field, as the goal is to yield an optimal correlation between the pre-op workup and the intraoperative findings.

The best surgical strategy for endometriosis still depends on the location, the extent of the lesions and the individual symptoms and needs of the patient. In contrast to oncological conditions, we may not be able to define stage-dependent algorithms. However, creating recommendations is still mandatory, since organ and function preservation should always be the focus of any surgical action. Not everything that is technically and surgically feasible is necessarily the best option for the patient: a good example is the current shift from the complete cystectomy of ovarian endometriomas to more preserving strategies such as sclerotherapy or argon plasma.

Laparoscopy has become the standard of care for the surgical treatment of endometriosis with significant patient benefits over open approaches. In recent years, robotically assisted techniques have proven to be another minimally invasive option. Cost and health economy issues as well as ergonomic considerations will fire the discussion about whether the laparoscopic approach will be replaced in the future.

With endometriosis being a hormone-dependent disease, medical treatment is still one relevant pillar of therapy. Combinations and gestagens with specific modifications remain the basic approach to date. It has to be shown whether other options that are based on the GnRH pathway or on the inhibition of oestrogens and local inflammatory mediators will find their way into clinical routine—the results from basic research are promising but sometimes lack a study design in human patients.

In summary, there is a lot to do to gain further insights into this complex disease. This journal issue is an important contribution to the present current knowledge for the improvement of diagnosis and care for endometriosis patients.

